# Sexual Dysfunction in Melanoma Survivors: A Cross-Sectional Study on Prevalence and Associated Factors

**DOI:** 10.3390/jcm14144891

**Published:** 2025-07-10

**Authors:** Daniel Muñoz-Barba, Manuel Sánchez-Díaz, Alejandro Molina-Leyva, Antonio Martínez-López, Salvador Arias-Santiago

**Affiliations:** 1 Dermatology Unit, Hospital Universitario Virgen de las Nieves, 18014 Granada, Spain; danielmb00w@gmail.com (D.M.-B.); alejandromolinaleyva@gmail.com (A.M.-L.); antoniomartinezlopez@aol.com (A.M.-L.); salvadorarias@ugr.es (S.A.-S.); 2 Dermatology Department, School of Medicine, University of Granada, 18016 Granada, Spain; 3 Melanoma Clinic, Hospital Universitario Virgen de las Nieves, 18014 Granada, Spain

**Keywords:** melanoma, quality of life, sexual dysfunction

## Abstract

**Background/Objectives**: Melanoma is a skin cancer that can lead to a poor prognosis. Unlike other oncologic diseases, there is scarce evidence regarding sexual function in melanoma patients, as well as factors associated with sexual dysfunction (SD). The aim of this study was to evaluate SD in a cohort of melanoma patients, as well as to describe associated factors. **Methods**: A cross-sectional analysis was conducted in individuals diagnosed with melanoma. Data regarding sociodemographic characteristics, clinical stage of the disease, quality of life, and sexual functioning were obtained through the use of validated assessment tools. The duration of the study was from 1 January 2023 to 1 January 2024. **Results**: Seventy-five patients were included. The mean age was 52.70 ± 14.07 years, and 61.33% (46/75) were females. Melanomas at stages III or IV comprised 18.67% (14/75) of the sample. A negative impact of the melanoma on sexual function was reported by 29.33% (22/75) of patients, with low sexual desire being the most frequent cause. Female SD was associated with older age, shorter disease duration, greater depression rates, and visible scar location after melanoma surgery (*p* < 0.05). Male SD correlated with higher anxiety and depression rates and worse quality of life (*p* < 0.05). No association was found for melanoma stage in any case (*p* > 0.30). **Conclusions**: Melanoma patients may suffer from SD, which can be associated with mood status disturbances, poor quality of life, and older age. Since the most frequent causes of a negative impact on sexuality are a reduction in sexual desire and the side effects of melanoma surgery, patients should be specifically asked about sexuality to improve holistic care of the disease, irrespective of disease stage.

## 1. Introduction

Cutaneous melanoma is a skin cancer that is responsible for most of the deaths related to skin tumours [1]. Despite the development of novel therapies for treating patients with advanced stages, disease control still remains a challenge in some groups of patients [2,3].

Within the health-related quality of life (HRQOL), sexual function is a highly relevant but frequently overlooked domain in clinical dermatology care [4,5,6]. Sexual health may be compromised in cancer patients due to the physical, emotional, and social consequences of the disease, and melanoma is no exception. While impairments in HRQOL have been documented in patients with advanced melanoma, particularly in relation to treatment burden [7,8], less is known about the specific impact on sexual function. Importantly, sexual dysfunction (SD) may affect patients irrespective of cancer stage and may be mediated by factors such as mood disorders, altered body image, treatment side effects, and perceived loss of desirability or self-esteem.

Unlike other cancers with more established data—such as breast or colorectal cancer [9,10]—there is a significant paucity of research on sexual health in patients with melanoma [11]. The existing literature on HRQoL in patients with melanoma has predominantly focused on general well-being and psychological outcomes. In contrast, domains such as sexual health remain markedly underrepresented and insufficiently investigated. Available studies suggest that although many patients with early-stage melanoma maintain a globally preserved quality of life, subsets of individuals—particularly women, older patients, and those with visible surgical scars—may experience declines in sexual satisfaction and intimacy [12,13,14].

In patients with advanced melanoma, systemic treatments such as immune checkpoint inhibitors and targeted therapies have revolutionised survival outcomes. However, these interventions are associated with adverse events, including fatigue, endocrine disturbances, and psychological distress, which may secondarily impair sexual function. For example, hypogonadism in male patients treated with immunotherapy has been associated with erectile dysfunction and reduced libido, contributing to long-term dissatisfaction with sexual life [15,16]. Moreover, in rare instances of mucosal melanoma affecting the genital area, surgical management can have direct anatomical and functional consequences for sexual activity [17].

Despite growing interest in the broader psychosocial implications of melanoma, the impact of the disease on sexual health continues to be a frequently overlooked component of patient care. Difficulties in addressing this issue may stem from both patients’ reluctance to share concerns and the lack of routine inquiry by healthcare professionals. Recognising and understanding the prevalence and contributing factors of SD in individuals with melanoma could enable the identification of those at greater risk and help implement targeted, patient-centred strategies to support long-term well-being.

Therefore, the aims of this study are: (a) to assess the prevalence of SD in a cohort of patients diagnosed with melanoma; (b) to identify clinical and psychological factors potentially associated with SD; and (c) to evaluate the impact of SD on various dimensions of quality of life in this population.

## 2. Materials and Methods

Design: A cross-sectional study was conducted between 1 January 2023 and 1 January 2024, enroling patients diagnosed with melanoma across all disease stages. The primary objective was to determine the prevalence of SD and to investigate clinical and HRQOL variables potentially associated with an increased risk of SD.

Patients: Patients included in the study were recruited sequentially through the Melanoma Unit of the Dermatology Department of a third-level hospital. This unit collects all cases of melanoma diagnosed and followed up at the hospital. Patients were asked to complete a questionnaire regarding HRQOL and SD.

Inclusion criteria: Participants were eligible if they met the following conditions: (a) a histologically confirmed diagnosis of melanoma, regardless of tumour stage or treatment status; (b) age ≥ 18 years; (c) current sexual activity, defined as engagement in sexual intercourse or manual sexual stimulation; and (d) provision of written informed consent for study participation.

Exclusion criteria: Patients were excluded if they: (a) declined to participate in the study, or (b) had any comorbid condition with a significant impact on overall quality of life.

Ethics: This study received approval from the Research Ethics Committee of Hospital Universitario Virgen de las Nieves and was conducted in full compliance with the principles outlined in the Declaration of Helsinki.

### 2.1. Main Variables

The main variables included those related to SD and HRQOL:

(a) International Index of Erectile Function (IIEF-5) [18] and Female Sexual Function Index (FSFI-6) [19] questionnaires: The IIEF-5 encompasses five key domains of male sexual health, with scores ≤ 21 indicating the presence of dysfunction. The FSFI-6 assesses six core dimensions of female sexual function, with a threshold score of ≤19 suggestive of SD.
(b) Patients were asked about the impact of the disease in terms of sexuality. Those patients who answered that melanoma had a negative impact were asked to describe the causes of the negative impact.
(c) Numeric Rating Scale (NRS) for sexual impairment: To assess perceived sexual impairment related to melanoma, patients were asked to rate the impact ranging from 1 to 10 [20].
(d) Dermatology Life Quality Index (DLQI): This instrument serves as a general indicator of dermatology-related quality of life for individuals aged 16 years and older. It comprises 10 items, each rated on a 4-point Likert scale ranging from 0 (no impact) to 3 (maximum impact), yielding a total score between 0 and 30. The items assess the impact of skin disease over the preceding seven days [21].

### 2.2. Secondary Variables: Other Variables of Interest Included

(a) The Hospital Anxiety and Depression Scale (HADS) was used to assess the presence of anxiety and depression. It consists of two subscales, one for anxiety and another for depression. A score ≥ 8 on any of the subscales was considered indicative of anxiety or depression, respectively [22].
(b) Variables related to the severity and characteristics of the disease:
  - TNM stage for melanoma as recommended by the 8th American Joint Committee on Cancer (AJCC) [23].
  - Age of onset, evolution time of the disease, date of diagnosis, location of the melanoma, and current and past treatments were collected.

### 2.3. Statistical Analysis

Descriptive statistics were employed to characterise the study population. Continuous variables are presented as means with standard deviations, whereas categorical variables are reported as absolute and relative frequencies. Group comparisons for categorical variables were conducted using the χ^2^ test or Fisher’s exact test, as appropriate. For comparisons involving continuous variables, either the Student’s *t*-test or the Wilcoxon–Mann–Whitney U test was applied based on distributional assumptions. To identify potential associated factors, simple linear regression analyses were performed for continuous predictors, reporting β coefficients and corresponding SDs. Statistical significance was set at *p* < 0.05. All analyses were conducted using JMP software, version 14.1.0 (SAS Institute, Cary, NC, USA).

## 3. Results

### 3.1. Sociodemographic and Clinical Characteristics of the Sample

A total of 75 patients diagnosed with cutaneous melanoma were included. The mean age was 52.7 years (±14.1), with a predominance of female participants (61.3%, 46/75). Most patients were in a relationship at the time of the study (77.3%, 58/75), and 54.7% (41/75) were actively employed. Regarding education, 34.7% had completed university studies, while 26.7% had only a basic education. Melanoma was most frequently located on the trunk (57.3%) and lower limbs (30.6%). The majority of the cohort (81.3%, 61/75) had early-stage disease (stage I–II), whereas 18.7% (14/75) had been diagnosed with stage III or IV melanoma. The median disease duration at the time of survey completion was 3.26 years (±6.77). Notably, 41.3% of participants had visible scarring from melanoma surgery. A detailed distribution of these characteristics is shown in Table 1.

### 3.2. Impact of Melanoma on Perceived Sexual Function

**Figure 1:**
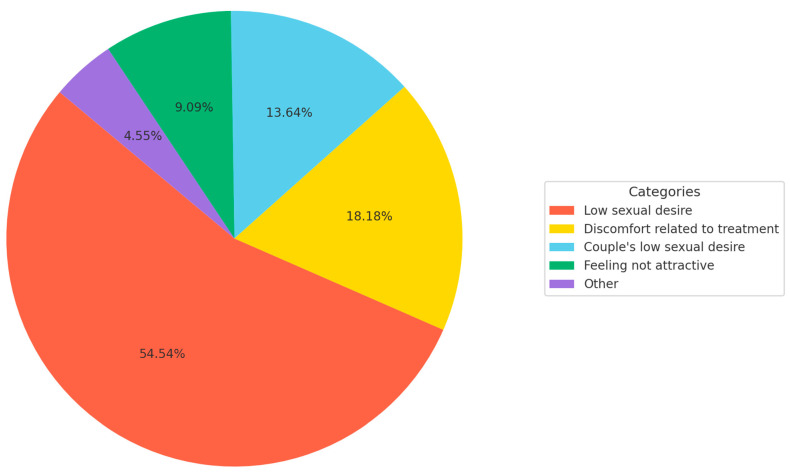
Primary causes of sexual dysfunction reported by patients with melanoma. The most commonly identified factor was low sexual desire (54.54%), representing more than half of the reported cases.

When directly asked about the influence of melanoma on their sexual health, 29.3% of patients (22/75) acknowledged a negative impact. Among these, the most frequently cited reason was a reduction in sexual desire (54.5%, 12/22), followed by physical discomfort associated with surgical sequelae or treatment (18.2%, 4/22), and diminished sexual desire in their partner (13.6%, 3/22). The mean score on the numeric rating scale (NRS) evaluating perceived sexual impairment was 2.25 (±2.95). Detailed distributions of these characteristics are shown in Table 1 and Figure 1.

### 3.3. Objective Assessment of Sexual Dysfunction

Among women (n = 46), the mean FSFI score was 17.8 (±9.46), with 41.3% (19/46) meeting the diagnostic threshold for SD (score ≤ 19). In men (n = 29), the mean IIEF-5 score was 17.65 (SD 5.67), and 68.9% (20/29) were classified as having SD (score ≤ 21). Overall, 52.0% (39/75) of the cohort presented with clinically significant SD based on these measures. The prevalence was significantly higher in men than in women (*p* < 0.01). A detailed distribution of these characteristics is shown in Table 1.

### 3.4. Factors Associated with Sexual Dysfunction

Univariate analyses were conducted to explore the relationship between clinical and psychosocial variables and the presence of SD, as defined by FSFI, IIEF, and NRS scores. A detailed distribution of these characteristics is shown in Table 2.

#### 3.4.1. Female Patients

In women, lower FSFI scores—indicating poorer sexual function—were significantly associated with older age (*p* < 0.001), older age at melanoma diagnosis (*p* < 0.001), shorter disease duration (*p* = 0.03), presence of visible surgical scars (*p* = 0.05), single marital status (*p* < 0.001), and higher scores for depression on the HADS scale (*p* = 0.03). No statistically significant associations were found for educational level, melanoma stage and location, performance of selective sentinel node biopsy, or DLQI scores (*p* > 0.40).

#### 3.4.2. Male Patients

Among men, lower IIEF scores were associated with elevated anxiety (*p* = 0.03), higher depression scores (*p* = 0.04), and lower DLQI scores (*p* = 0.04). Sexual function did not correlate with age, educational level, marital status, age at onset and disease duration, melanoma stage and location, performance of selective sentinel node biopsy, or scar visibility in male patients (*p* > 0.10).

#### 3.4.3. Global Perceived Impairment

The NRS, used to assess self-reported global impairment in sexual function, revealed that higher levels of perceived dysfunction were associated with older age (*p* < 0.01), shorter disease duration (*p* = 0.05), worse DLQI scores (*p* = 0.01), and greater levels of anxiety and depression (*p* < 0.01). Similar to the results from objective indices, no significant associations were observed for melanoma stage and location, performance of selective sentinel node biopsy, presence of visible scar, educational level, and marital status (*p* > 0.10).

## 4. Discussion

This study provides novel and clinically relevant evidence on the sexual health of patients with cutaneous melanoma, a domain that has been studied in other dermatological and oncological diseases [24,25,26]. Our findings demonstrate that over half of the patients evaluated met the criteria for SD, with a significant proportion also reporting subjective impairment attributable to melanoma. These data underscore the importance of explicitly assessing sexual health as part of the holistic management of melanoma survivors.

SD was more prevalent in men (68.9%) than in women (41.3%), consistent with findings in the general population and among individuals with other dermatologic conditions [27,28]. In line with previous research, the most commonly reported complaint was a decline in sexual desire, which often reflects a complex interplay between biological, psychological, and relational factors [29]. Notably, both objective measures (IIEF/FSFI) and patient-reported outcomes (NRS) consistently identified depression, anxiety, and impaired quality of life as significant correlates of SD. These associations highlight the multifactorial nature of sexual impairment and reinforce the established interdependence between mood status and sexual function in cancer populations [30,31].

Moreover, this impact on mood status in patients with melanoma has already been described in previous studies, although their primary aim was not to quantify the effect on sexual health. These studies demonstrated that higher stages at diagnosis, older age, and lower educational level were associated with reduced HRQOL [13]. Additionally, mood disturbances such as anxiety and depression have been recently examined in this population, with female gender and lower educational attainment increasing the likelihood of emotional distress [14]. According to our findings, SD may also be linked to mood disturbances and age, but does not appear to be associated with educational level.

Our analysis also reveals important gender-specific differences in the profile and predictors of SD. In female patients, older age, visible scarring, and being single were prominent risk factors. The association between body image disturbances and sexual distress has been described in women with other malignancies, such as breast cancer [32], but has not been previously quantified in melanoma. The presence of visible scars—often on highly exposed anatomical sites—may affect self-perception, sexual confidence, and emotional intimacy. In contrast, male SD was more strongly associated with psychological distress (anxiety, depression) and DLQI impairment, echoing the pattern observed in studies of sexual health impairment secondary to other dermatological chronic diseases [28]. These distinctions suggest that targeted interventions should be adapted to gender-specific vulnerabilities.

Importantly, we observed no significant correlation between melanoma stage and either objective or subjective measures of SD. This finding contrasts with studies in other cancer types, where advanced disease often predicts greater sexual impairment [9,10]. In melanoma, however, it may be that functional and psychological sequelae arise not only from disease severity but also from treatment side effects, fear of recurrence, and broader psychosocial disruptions. Despite these risks, sexual health is rarely addressed during follow-up, reflecting a persistent gap in survivorship care [33].

This study’s strengths include the use of validated instruments for sexual function and mood assessment, the inclusion of both male and female patients across all disease stages, and the prospective collection of data in a real-world clinical setting. Nonetheless, several limitations must be acknowledged. The lack of a control group precludes comparisons with the general population, and the sample size limits the power of subgroup comparisons. In addition, the cross-sectional design does not allow for longitudinal assessment of SD evolution or its causal relationships with treatment or psychosocial variables. Future studies should incorporate larger, multi-centre cohorts and adopt a prospective design to better capture the temporal dynamics of sexual health in melanoma.

Taken together, our findings provide compelling evidence that sexual health concerns are both prevalent and clinically meaningful among patients with melanoma. Routine assessment of sexual function, especially among older individuals, those with visible scarring, and those experiencing mood disturbances, may allow for early identification of vulnerable patients and implementation of supportive interventions such as psycho-oncology, sexual counselling, or endocrinological evaluation. Additionally, educational efforts are needed to empower clinicians to discuss sexual health openly and to normalise these conversations within the standard of melanoma care.

## 5. Conclusions

SD is a common and under-recognised problem among patients with melanoma, affecting more than half of those assessed in this study. Our results reveal that SD is not associated with melanoma stage, but rather with age, mood disturbances, body image concerns, and perceived quality of life. These findings highlight the need for a patient-centred approach that incorporates sexual health evaluation into routine clinical practice. Future research should aim to characterise the longitudinal course of SD in melanoma, identify modifiable risk factors, and develop targeted interventions to mitigate its impact on survivorship and emotional well-being.

## Figures and Tables

**Table 1:** Distribution of sociodemographic characteristics, melanoma-related clinical variables, and key indicators of quality of life, mood, and sexual function in patients with melanoma.

Distribution of Sociodemographic Characteristics, Melanoma-Related Clinical Variables, and Key Indicators of Quality of Life, Mood, and Sexual Function in Patients with Melanoma (N = 75)					
**Socio-demographic features**					
Age (years)	52.70 ± 14.07		Marital status	With couple	77.33% (58/75)
				Single	22.67% (17/75)
Sex (%)	Male:	38.67% (29/75)	Occupation	Employed	54.67% (41/75)
	Female:	61.33% (46/75)		Unemployed	45.33% (34/75)
Educational level	Basic education	26.67% (20/75)	Professional education		17.33% (13/75)
	Secondary education	21.33% (16/75)	University education		34.67% (26/75)
**Disease characteristics**					
Disease duration (years)	3.26 ± 6.77		Visible location of scar	41.33% (31/75)	
Melanoma stage	I–II	81.33% (61/75)	Melanoma location	Trunk	57.33% (43/75)
				Upper limbs	4.00% (3/75)
				Lower limbs	30.67% (23/75)
	III–IV	18.67% (14/75)		Face	8.00% (6/75)
**Quality of life indicators**					
DLQI	3.60 ± 3.95		Negative impact of the disease on sexuality (%)	29.33% (22/75)	
HADS Depression (score)	2.34 ± 2.98		HADS Anxiety (score)	3.88 ± 4.16	
FSFI (% of female sexual dysfunction)	41.30% (19/46)		IIEF (% of male sexual dysfunction)	68.96% (20/29)	
Overall sexual dysfunction	52.00% (39/75)		NRS for sexual dysfunction	2.25 ± 2.95	

DLQI: Dermatology Quality of Life Index; HADS: Hospital Anxiety and Depression Score; FSFI: Female Sexual Function Index; IIEF: International Index of Erectile Function; NRS: Numeric Rating Scale.

**Table 2:** Univariate Analyses of Factors Associated with Sexual Dysfunction in Melanoma Patients.

Univariate Analyses of Factors Associated with Sexual Dysfunction in Patients with Melanoma. Associations Were Explored for Female Sexual Dysfunction (FSFI Scores), Male Sexual Dysfunction (IIEF Scores), and Global Sexual Dysfunction (Measured by the Numeric Rating Scale, NRS)						
Factors	Female sexual dysfunction (FSFI)		Male sexual dysfunction (IIEF)		Global sexual dysfunction (NRS for SD)	
	Mean/%/beta	*p* value	Mean/%/beta	*p* value	Mean/Beta	*p* value
Age (years)	−0.32 ± 0.08	<0.01	−0.06 ± 0.10	0.53	0.06 ± 0.02	<0.01
Educational level	Basic: 17.05 ± 2.13	0.64	Basic: 18 ± 1.44	0.72	Basic: 2.52 ± 0.49	0.44
	Superior: 18.38 ± 1.87		Superior: 17.23 ± 1.59		Superior: 2 ± 0.47	
Marital status	Couple: 20.02 ± 1.46	<0.01	Couple: 17.73 ± 1.20	0.78	Couple: 2.48 ± 0.38	0.21
	Single: 10.72 ± 2.61		Single: 17.33 ± 2.35		Single: 1.47 ± 0.71	
Age at onset of disease (years)	−0.30 ± 0.08	<0.01	−0.02 ± 0.07	0.76	0.04 ± 0.02	0.07
Disease duration (months)	0.11 ± 0.04	0.01	0.03 ± 0.02	0.23	−0.01 ± 0.01	0.05
Melanoma stage	I–II: 17.71 ± 1.53	0.88	I–II: 18.71 ± 2.17	0.57	I–II: 2.14 ± 0.37	0.52
	III–IV: 18.28 ± 3.61		III–IV: 17.31 ± 1.22		III–IV: 2.71 ± 0.79	
Visible scar location	Yes: 15.84 ± 1.86	0.05	Yes: 16 ± 2.33	0.43	Yes: 1.93 ± 0.53	0.43
	No: 20.14 ± 2.03		No: 18.08 ± 1.19		No: 2.47 ± 0.44	
Sentinel lymph node biopsy performed	Yes: 73.68% (14/19)	0.44	Yes: 65% (13/20)	0.48	Yes: 66.67% (24/36)	0.52
	No: 26.32% (5/19)		No: 35% (7/20)		No: 33.33% (12/36)	
Melanoma location (Face/Lower limbs/Upper Limbs/Trunk)	Yes: 5.26%/36.84%/5.26%/52.63%	0.45	Yes: 15%/5%/5%/75%	1.0	Yes: 10.26%/20.51%/5.13%/64.10%	0.23
	No: 3.70%/55.56%/0%/40.74%		No: 11.11%/0%/11.11%/77.78%		No: 5.56%/41.67%/2.78%/50%	
Anxiety (HADS-A ≥ 8)	Yes: 19.25 ± 2.75	0.54	Yes: 9 ± 5.52	0.03	Yes: 4.76 ± 0.75	<0.01
	No: 17.29 ± 1.63		No: 17.96 ± 1.04		No: 1.75 ± 0.34	
Depression (HADS-D ≥ 8)	Yes: 1 ± 9.21	0.03	Yes: 9 ± 5.52	0.04	Yes: 7 ± 2.02	0.02
	No: 18.17 ± 1.37		No: 17.96 ± 1.04		No: 2.12 ± 0.33	
DLQI	−0.22 ± 0.23	0.50	−1.00 ± 0.53	0.04	0.21 ± 0.08	0.01

HADS: Hospital Anxiety and Depression Score; DLQI: Dermatology Quality of Life Index.

## Data Availability

The data that support the findings of this study are available from the corresponding author upon reasonable request.

## Abbreviations

The following abbreviations are used in this manuscript:

SD	Sexual Dysfunction
HRQOL	Health-related quality of life
IIEF-5	International Index of Erectile Function
FSFI-6	Female Sexual Function Index
NRS	Numeric Rating Scale
DLQI	Dermatology Life Quality Index
HADS	Hospital Anxiety and Depression Scale
AJCC	American Joint Committee on Cancer
